# *Corylus avellana* “Nocciola Piemonte”: metabolomics focused on polar lipids and phenolic compounds in fresh and roasted hazelnuts

**DOI:** 10.3389/fpls.2023.1252196

**Published:** 2023-10-11

**Authors:** Antonietta Cerulli, Assunta Napolitano, Beata Olas, Milena Masullo, Sonia Piacente

**Affiliations:** ^1^ Department of Pharmacy, University of Salerno, Fisciano, SA, Italy; ^2^ Department of General Biochemistry, Institute of Biochemistry, Faculty of Biology and Environmental Protection, University of Lodz, Lodz, Poland; ^3^ National Biodiversity Future Center (NBFC), Palermo, Italy

**Keywords:** *Corylus avellana*, “Nocciola Piemonte” PGI, fresh and roasted hazelnut, polar lipids, phenolic compounds, LC-ESI/LTQOrbitrap/MS/MS^n^, antioxidant activity

## Abstract

The common hazel plant (*Corylus avellana* L., Betulaceae) is one of the most popular tree nuts widespread in Europe and Asia. In Italy, there are different cultivars among which the cultivar affording the valuable hazelnut “Tonda Gentile Trilobata,” also known as “Tonda Gentile delle Langhe,” covered by the Protected Geographical Indication (PGI) label “Nocciola Piemonte” (NP), known for its sweetness, cooked-bread aroma, and the low intensity of the burnt aroma. In order to obtain a detailed and in-depth characterization of the polar fraction of fresh (NPF) and roasted (NPR) kernels of NP the analysis of the *n*-butanol extracts by liquid chromatography coupled to electrospray ionization and high-resolution mass spectrometry (LC-ESI/HRMS) was carried out. Moreover, to evaluate the quantitative distribution of the most representative polar lipids in NPF and NPR, the analysis by liquid chromatography combined with tandem mass spectrometry (LC-MS/MS) was performed. To unambiguously identify the phenolic compounds highlighted by the LC-ESI/HRMS profiles, they were isolated from the *n*-butanol extract and characterized by Nuclear Magnetic Resonance (NMR) experiments. Finally, the ability of the isolated compounds to exert radical scavenging activity and to inhibit the lipid peroxidation induced by H_2_O_2_ or H_2_O_2_/Fe^2+^ was tested by Trolox Equivalent Antioxidant Capacity (TEAC) and thiobarbituric acid reactive substances (TBARS) assays, respectively. The LC-ESI/HRMS allowed to ascertain the presence of phenolic compounds and multiple classes of polar lipids including phospholipids, glycolipids, sphingolipids, and oxylipins. The quantitative analysis highlighted in NPR fraction a lipid content three times higher than in NPF, evidencing lyso-phospholipids and phospholipids as the most represented lipid classes in both NPF and NPR, together accounting for 94 and 97% of the considered lipids, respectively. Furthermore, phytochemical analysis permitted to identify flavonoid and diarylheptanoid derivatives. In particular, quercetin 3-O-β-D-galactopyranosyl-(1→2)-β-D-glucopyranoside and myricetin-3-O-α-L-rhamnopyranoside showed the highest antioxidant activity, exhibiting TEAC values similar to that of quercetin, used as reference compound (2.00 ± 0.03 and 2.06 ± 0.03 mM vs 2.03 ± 0.03 mM, respectively). Moreover, most of the tested compounds were found to reduce lipid peroxidation induced by H_2_O_2_ and H_2_O_2_/Fe^2+^ more than curcumin used as positive control, with myricetin-3-O-α-L-rhamnopyranoside determining 44.4 % and 34.1 % inhibition percentage, respectively.

## Introduction

The common hazel plant (*Corylus avellana* L., Betulaceae) is one of the most popular tree nuts, widespread in Europe and Asia. It grows in mild climates such as Turkey, Spain, and Italy. Turkey represents the leader country in the production and exportation of hazelnuts, accounting for more than 70% of the world crop, followed by Italy (15%–20%) and the United States (less than 5%) (Caligiani et al., 2014).

Currently, in Italy, there are two hazelnut varieties registered by the European Union with the Protected Geographical Indication (PGI) label, that is, “Nocciola di Giffoni” and “Nocciola Piemonte” (also known as “Tonda Gentile Trilobata” or “Tonda Gentile delle Langhe”). Both are widely recognized as excellent hazelnuts for industrial processing into roasted kernels (Caligiani et al., 2014).

Our previous investigations focused the attention on the leaves, flowers, shells, green leafy involucres, and kernel of *C. avellana* cultivar “Tonda di Giffoni,” leading to the isolation of natural compounds belonging to the class of flavonoids, caffeic acids, cyclic diarylheptanoids, and cyclic diaryletherheptanoids, named giffonins A–X (Masullo et al., 2015a; Masullo et al., 2015b; Masullo et al., 2016; Cerulli et al., 2017; Masullo et al., 2017; Cerulli et al., 2018b; Cerulli et al., 2018c; Napolitano et al., 2018; Bottone et al., 2019; Masullo et al., 2021b; Masullo et al., 2022).

The edible part of hazelnuts is kernel, a food abundantly consumed raw or roasted, appreciated for flavor and texture. As the dry heat treatment of kernels leads to the development of the flavor, color, and crunchy texture (Slatnar et al., 2014; Binello et al., 2018), confectionary industry prefers roasted hazelnuts to produce chocolates, ice creams, and a wide variety of desserts (Jiang et al., 2021; Cristofori et al., 2022).Several research groups have reported the benefits of the inclusion of nuts in the human diet. Among nut species, US Food and Drug Administration (FDA) has recognized hazelnuts as “heart-healthy” foods by virtue of their nutritional and nutraceutical properties (Alasalvar and Pelvan, 2011).

The beneficial effects of hazelnuts on human health are related to the content of monounsaturated (MUFA) and polyunsaturated fatty acids (PUFAs), proteins, carbohydrates, dietary fibers, phytosterols (mainly β-sitosterol), vitamins (vitamin E), antioxidant phenolics, and minerals. MUFA and PUFA are responsible for health benefits including the lowering of plasma oxidized Low Density Lipoprotein (LDL) levels (Sun et al., 2022) and the prevention of cardiovascular diseases, diabetes, cancer, Alzheimer’s disease, and dementia (Rincón-Cervera et al., 2022), while tocopherols of hazelnuts are reported to exert positive effects in preventing heart disease and various types of cancer by inhibiting tumor growth and enhancing the human immune system (Dietrich et al., 2006; Bacchetta et al., 2013). Hazelnuts also contain several phytosterols; they constitute their cell membranes, stabilizing the phospholipid bilayer. In the human gut, due to their high hydrophobicity, phytosterols interfere with cholesterol absorption, consequently contributing to the control of cholesterol levels (Rondanelli et al, 2023, Ostlund, 2002; Sabaté and Salas-Salvadó, 2006).

Studies on *C. avellana* “Nocciola Piemonte” focused the attention mainly on volatile organic compounds present in hazelnuts (Ortega-Gavilán et al., 2023) and on the investigation of hazelnut primary metabolome, with particular attention to monounsaturated and polyunsaturated fatty acids (Caligiani et al., 2014; Granata et al., 2017; Cialiè Rosso et al., 2021). Moreover, their phenolic compound profile was also investigated, highlighting changes caused by different storage conditions (Ghirardello et al., 2016).

Noteworthy, our investigation on “Nocciola di Giffoni” hazelnuts highlighted the occurrence of polar lipids belonging to the class of phospholipids, glycolipids, sphingolipids, and oxylipins, reported in the literature for their biological activity (Cerulli et al., 2021). Oxylipins are involved in *in-vivo* inflammatory cascades, pain perception, and skin barrier integrity, whereas, phospholipids counter the declining of memory, prevent the development of nonalcoholic fatty liver disease, and are involved in the mechanism of angiogenesis, inflammation, cancer, and diabetes (Inoue et al., 2011; Kihara et al., 2015; Napolitano et al., 2018). Glycolipids have been reported to display antiviral, antitumor, and anti-inflammatory activities (Napolitano et al., 2007; Napolitano et al., 2018). Based on the interesting activity reported for these polar lipids (Cannavacciuolo et al., 2022), and considering that, in literature, there was no comprehensive information about the polar lipid composition of “Nocciola Piemonte” hazelnuts, an in-depth investigation on polar metabolites occurring in “Nocciola Piemonte” kernels was carried out, with a special focus on phenolics and polar lipids, to fill the gap existing in “Nocciola Piemonte” literature on the latter metabolites. Considering that hazelnuts are generally consumed roasted, and there is evidence that the roasting process could influence their chemical composition (Locatelli et al., 2015), both fresh (NPF) and roasted (NPR) “Nocciola Piemonte” kernels (without skin) were considered. Thereby, by following an analytical approach based on high-performance liquid chromatography coupled to linear ion-trap, allowing multiple levels of fragmentation, and orbitrap high-resolution mass spectrometry with both negative and positive electrospray ionization [LC-(±)ESI/LTQOrbitrap/MS/MS^n^], exhaustive and thorough profiles of the *n*-butanol extract of both NPF and NPR were achieved. In particular, the presence of several lipid classes including phospholipids, glycolipids, sphingolipids, and oxylipins, along with different phenolic derivatives was ascertained. Subsequently, with the aim to obtain a quantitative distribution of the main polar lipid classes both in NPF and NPR, LC-ESI/QTrap/MS/MS analysis by Multiple Reaction Monitoring (MRM) experiments was carried out on the *n*-butanol extracts.

Moreover, to obtain an unequivocal structural characterization of phenolics highlighted by LC-ESI/HRMS analysis, the *n*-butanol extract of NPR kernel was investigated, leading to the isolation and structural identification, by 1D- and 2D-NMR experiments, of metabolites belonging to flavonoid and diarylheptanoid classes.

Finally, in order to evaluate the isolated compounds for their antioxidant activity and inhibitory effects on human plasma lipid peroxidation induced by H_2_O_2_ and H_2_O_2_/Fe^2+^, a spectrophotometric Trolox Equivalent Antioxidant Capacity (TEAC) assay determining the radical-scavenging activity and a test measuring the concentration of thiobarbituric acid reactive substances (TBARS) were performed, respectively.

## Material and methods

### Chemicals and reagents

Chloroform, *n*-hexane, methanol (MeOH), and *n*-butanol were purchased from VWR (Milan, Italy). Water, acetonitrile (ACN), 2-propanol (IPA), and formic acid used for LC-MS were of Merck brand and were bought from Deltek (Naples, Italy); methanol-*d*_4_ (99.95%) was bought by Sigma-Aldrich (Milan, Italy).

The following polar lipids of Avanti Polar Lipids brand (bought from Merck, Darmstadt, Germany) were used as internal reference standards: phosphatidylglycerol PG (8:0;8:0), 10 µg/ml; phosphatidylcholine PC (8:0;8:0), 30 µg/ml; phosphatidylethanolamine PE (8:0;8:0), 60 µg/ml; phosphatidic acid PA (14:0;14:0), 20 µg/ml; phosphatidylserine PS (14:0;14:0), 2.5 µg/ml; sulfoquinovosyldiacylglycerol SQDG (18:3;16:0), 20 µg/ml; monogalactosyldiacylglycerol MGDG (18:3;18:3), 50 µg/ml. Dimethylsulfoxide (DMSO), thiobarbituric acid (TBA), and H_2_O_2_ were purchased from Sigma-Aldrich (St. Louis, MO., USA).

### Samples

*Corylus avellana* L. hazelnuts (“Nocciola Piemonte”) were obtained from la Gentile s.r.l. (N:44°34’38.6”, E:8°11’42.7”). Hazelnuts (size in the range of 17 mm–21 mm) were collected in August 2021. A voucher specimen was deposited in Department of Pharmacy, University of Salerno, Italy.

### Extraction workflow

*C. avellana* kernels were crushed by a knife and stored at room temperature for 3 days. Hazelnuts (220 g), after removal of the skin, were submitted to defatting with *n*-hexane and chloroform, and successively extracted with MeOH, each time using 4.5 L of solvent. The extractions were repeated, every 3 days, until, for each solvent, the last recovery was less than 10%. The MeOH extract was filtered and dried under vacuum, then, in order to remove oily compounds (e.g., triglycerides), it was partitioned using *n*-hexane and MeOH. Finally, with the aim to remove the free sugars that interfere with the identification of primary and specialized metabolites, the derived MeOH extract underwent to *n*-butanol/water partition, thus obtaining 8.0 g of *n*-butanol extract. Hazelnut kernels without skin (250 g) were roasted as previously reported by Napolitano et al. (Cerulli et al., 2018c; Napolitano et al., 2018) and extracted in the same way of fresh hazelnuts to obtain 9.1 g of *n*-butanol extract.

### LC-ESI/HRMS/MS^n^ qualitative analysis

The analysis of both NPF and NPR *n*-butanol extracts was performed using LC-ESI/HRMS instrument, parameters, and conditions described by Napolitano et al. (Napolitano et al., 2018). Xcalibur software version 2.1 was used for instrument control, data acquisition, and data analysis. For each sample, three replicates were performed.

### LC-ESI/MS/MS quantitative analysis

The analysis was performed using a LC-ESI/QTrap/MS System consisting of a Shimadzu Nexera X2 UPLC system in line with a Linear Ion Trap Quadrupole mass spectrometer (QTRAP 6500) (ABSciex, Foster City, CA, USA) in negative ionization mode. A Kinetex EVO C18 column (Phenomenex, 100 mm × 2.1 mm i.d., 1.7 μm), kept at 40°C, and a combination of A (60:40 water:ACN, v/v) and B (85:10:5 IPA:ACN:water, v/v), both containing 0.1% (v/v) formic acid, as mobile phase, were employed for the chromatographic separation. A linear gradient from 15% to 60% B in 9.33 min, to 90% B in 10 min, to 100% B in 4.67 min, held to 100% B for 6 min, back to 15% in 1.3 min, and a flow rate of 0.3 mL/min were used. Six microliter of each extract (0.5 mg/mL) added of internal reference standard lipids was injected. For Ion-Spray operation, the following experimental conditions were used: curtain gas (CUR) = 40; Ion-Spray voltage (IS) = −4500; source temperature (TEM) = 350°C; ion source gas 1 (GS1) = 25; ion source gas 2 (GS2) = 25.

The eluate from the chromatographic column was monitored by MS/MS in MRM mode, by using lipid-class specific values for declustering potential, focusing potential, entrance potential, collision energy, and collision cell exit potential. Three replicates of each sample were performed. The chromatographic peak areas of detected lipids in each sample, contained in the data set generated by the software supplied by the manufacturer (Analyst 1.6.2), were used as quantitative response. Normalization of the data was performed, calculating the ratio between the peak area of each lipid and that of the corresponding internal reference standard (López-Bascón et al., 2020).

Recovery experiments were performed by adding reference standard solutions at low-, medium-, and high-concentration range in a known amount of both NPF and NPR samples and subsequently extracted as above described and analyzed by LC-ESI/QTrap/MS/MS. Good recovery values (%) in the range of 92% and 108% were obtained.

### Isolation of phenolics and NMR analysis

Three grams of *n*-butanol extract of roasted hazelnuts were fractionated on a Sephadex LH-20 (Pharmacia) column (100 cm × 5 cm), using 100% MeOH as mobile phase. In this way, 52 fractions were obtained and monitored by thin-layer chromatography (TLC). To isolate pure compounds, some fractions were chromatographed by semipreparative isocratic RP-HPLC on a Waters 590 system (Waters R401 refractive index detector) a Rheodyne injector, and a Waters XTerra Prep MSC18 column (300 mm × 7.8 mm i.d.), using MeOH-H_2_O (2:3) as mobile phase; a flow rate of 2.5 mL/min was used.

By this way, fractions 20–27 (31.0 mg) yielded compounds **4** (2.1 mg, *R*_t_ = 9.2 min), **8** (1.6 mg, *R*_t_ = 18.2 min), and **11** (1.3 mg, *R*_t_ = 21.6 min); fractions 28–33 (21.4 mg) yielded compound **5** (2.3 mg, *R*_t_ = 24.0 min); fractions 34–38 (27.5 mg) yielded compound 3 (1.9 mg, *R*_t_ = 14.3 min); fractions 39–44 (30.1 mg) yielded compounds **10** (3.4 mg, *R*_t_ = 20.8 min) and **9** (2.1 mg, *R*_t_ = 24.2 min); fractions 45–47 (8.5 mg) yielded compound **6** (2.1 mg, *R*_t_ = 24.2 min). Fraction 50 corresponded to compound **7** (2.3 mg). The purity of these compounds (>99%) was determined by HPLC analysis (Masullo et al., 2015a; Masullo et al., 2015b; Masullo et al., 2016; Masullo et al., 2017).

NMR experiments were acquired in methanol-*d*_4_ on a Bruker DRX-600 spectrometer (Bruker BioSpin GmBH, Rheinstetten, Germany) equipped with a Bruker 5-mm TCI CryoProbe at 300 K. Data processing was carried out with Topspin 3.2 software.

### TEAC assays

Radical-scavenging activity was determined by spectrophotometric TEAC assay according to previously described procedures(Maldini et al., 2011; Cerulli et al., 2018a). In particular, in the TEAC assay, the antioxidant activity of isolated compounds (range of 0.3 mM–1.5 mM) was expressed as TEAC value in comparison with the TEAC activity of the reference compound quercetin. The TEAC value is defined as the concentration of a standard Trolox solution with the same antioxidant activity of a 1 mM concentration of the tested compound (calibration equation for Trolox: *y* = 31.863× + 52.550, *R*_2_ = 1.00).

### Lipid peroxidation measurement

Lipid peroxidation was quantified by measuring the concentration of TBARS. Stock solutions of each compound were prepared in 50% DMSO. The final concentration of DMSO in the samples was lower than 0.05%. Fresh human plasma was obtained from medication-free, regular donors at the blood bank (Lodz, Poland). Samples of human plasma were incubated with phenolic compounds and curcumin at the final concentrations of 0.1 µM–100 µM (30 min, at 37°C) alone and plus 2 mM H_2_O_2_ (30 min, at 37°C) and at the final concentration of 10 µM plus 4.7 mM H_2_O_2_/3.8 mM Fe_2_SO_4_/2.5 mM EDTA. Pure compounds were tested by using the TBARS assay as previously reported (Masullo et al., 2015b; Masullo et al., 2016; Cerulli et al., 2017). Three independent experiments were carried out.

### Statistical analysis

The statistical analysis was done by several tests. The Q-Dixon test was performed to eliminate uncertain data. All the values in this study were expressed as mean ± standard deviation (SD). The statistical was carried out with one-way analysis of variance for repeated measurements. The statistically significant differences were also evaluated by applying the paired Student’s t-test. The significance level used was *p* < 0.05 unless otherwise indicated. Microsoft Excel 2016 was used for statistical analyses.

## Results and discussion

### LC-ESI/HRMS/MS^n^ analysis of “Nocciola Piemonte” hazelnuts

With the aim to define the chemical profile of the *n*-butanol extract of both fresh and roasted “Nocciola Piemonte” kernels, an analytical approach based on LC-ESI/LTQOrbitrap/MS/MS^n^ was carried out. In particular, both negative and positive electrospray ionizations were used considering that lipid classes differ in their ionization capacity, depending on structure and polarity. Furthermore, in addition to an RP-C18 column, an RP-C4 column was used to reduce the strong interactions with the stationary phase of high molecular weight molecules such as phospholipids (Napolitano et al., 2018; Cannavacciuolo et al., 2022).

The careful analysis of accurate masses, characteristic fragmentation patterns, chromatographic behavior, and literature data allowed to putatively ascertain the occurrence, in both NPF and NPR extracts, of polar lipids belonging to oxylipins, phospholipids, sphingolipids, and glycolipids, as well as of phenolic derivatives mainly belonging to flavonoid and diarylheptanoid classes (**Tables 1**, **2**).

**Table 1:** Polar lipids and phenolic compounds putatively identified in fresh and roasted “Nocciola Piemonte” extracts by using a RP-C18 column for the chromatographic separation.

n°	Compound	*R_t_* (min)	Molecular Formula	[M-H]^-^	[(M+FA)-H]^-^	[M+H]^+^	Error (ppm)	Characteristic product ions	NPR	NPF
**1**	sucrose	1.94^a^	C_12_H_22_O_11_		387.1149		3.61	179, 143	+	+
**2**	glycerophosphorylinositol	2.13^b^	C_9_H_19_O_11_P	333.0592			3.23	259, 241, 153	+	+
**3**	quercetin 3-*O*-β-D-galactopyranosyl-(1→2)-β-D-glucopyranoside*	5.74^a^	C_27_H_30_O_17_	625.1399			0.04	463, 343, 301	+	+
**4**	giffonin P	6.13^a^	C_19_H_22_O_7_	361.1286			0.45	343, 301, 271, 241	+	**-**
**5**	kaempferol 3-*O*-β-D-glucopyranosyl-(1→2)-β-D-glucopyranoside*	6.25^a^	C_27_H_30_O_16_	609.1448			-0.28	447, 327, 285	+	**-**
**6**	myricetin-3-*O*-α-L-rhamnopyranoside*	6.54^a^	C_21_H_20_O_12_	463.0870			0.07	317	+	–
**7**	quercetin 3-*O*-β-D-glucopyranoside*	6.70^a^	C_21_H_20_O_12_	463.0867			0.64	343, 301	+	+
**8**	giffonin L	6.74^a^	C_19_H_22_O_6_	345.1331			-0.14	327	+	–
**9**	kaempferol -3-*O*-β-D-glucopyranoside*	7.40^a^	C_21_H_20_O_11_	447.0923			0.06	327, 285	+	+
**10**	carpinontriol B	8.08^a^	C_19_H_20_O_6_	343.1181			0.48	325, 283, 269	+	+
**11**	giffonin V	9.65^a^	C_19_H_20_O_5_	327.1233			0.65	309, 283, 253	+	–
**12**	9,12,13-TriHOME (10)	10.34^b^	C_18_H_34_O_5_	329.2326			1.18	311, 293, 291, 229, 211, 199, 197, 171	+	+
**13**	l-PC (18:2-1O)	11.20^a^	C_26_H_50_O_8_NP		580.3245		-0.29	520, 431, 295, 242	+	–
**14**	l-PC (18:2-1O)	11.36^a^	C_26_H_50_O_8_NP		580.3245		-0.29	520, 431, 295, 242	+	–
**15**	DGMG (18:3)	13.44^b^	C_33_H_56_O_14_	675.3593^b^	721.3649		0.68^b^	415, 397, 323, 235	+	+
**16**	DGMG (18:2)	14.38^b^	C_33_H_58_O_14_	677.3755^b^	723.3801		1.27^b^	415, 397, 279, 235	+	+
**17**	l-PC (16:1)	14.46^a^	C_24_H_48_O_7_NP		538.3138		-0.31	478, 253, 242	+	–
**18**	l-PC (16:1)	14.74^a^	C_24_H_48_O_7_NP		538.3141		0.38	478, 389, 253, 242	+	+
**19**	12,13-DiHOME (9)	15.12^b^	C_18_H_34_O_4_	313.2378			1.64	295, 277, 213, 183	+	+
**20**	DGMG (18:2)	15.13^b^	C_33_H_58_O_14_	677.3740^b^	723.3798		-0.26^b^	415, 397, 323, 279, 235	+	+
**21**	l-PE (18:2)	15.15^b^	C_23_H_44_O_7_NP	476.2771			-0.20	415, 279, 214, 196, 153	+	+
**22**	l-PC (18:2)	15.23^a^	C_26_H_50_O_7_NP		564.3298^a^	520.3362^d^	0.43^a^	504, 415, 279, 242, 224	+	+
**23**	9,10-DiHOME (12)	15.47^b^	C_18_H_34_O_4_	313.2381			2.44	295, 277, 269, 245, 201, 183, 171	–	+
**24**	DGMG (16:0)	15.66^b^	C_31_H_58_O_14_	653.3732^b^	699.3799		-1.11^b^	415, 397, 235	+	+
**25**	l-PE (18:2)	15.78^b^	C_23_H_44_O_7_NP	476.2778			1.27	415, 279, 214, 196, 153	+	+
**26**	l-PC (18:2)	15.85^a^	C_26_H_50_O_7_NP		564.3299^a^	520.3369^d^	0.54^a^	504, 415, 279, 242, 224	+	+
**27**	l-PE (16:0)	16.63^b^	C_21_H_44_O_7_NP	452.2772			0.19	391, 255, 214, 196, 153	+	+
**28**	DGMG (16:0)	16.67^b^	C_31_H_58_O_14_	653.3745^b^	699.3798		0.23^b^	415, 397, 255, 235	+	+
**29**	9,10-DHSA	16.76^b^	C_18_H_36_O_4_	315.2535			1.76	297, 279, 201, 187, 171	+	+
**30**	l-PC (16:0)	16.79^a^	C_24_H_50_O_7_NP		540.3290		-1.03	480, 391, 255, 242, 224	+	+
**31**	DGMG (18:1)	17.11^b^	C_33_H_60_O_14_	679.3888^b^	725.3954		-1.08^b^	415, 397, 281, 235	+	+
**32**	l-PE (16:0)	17.57^b^	C_21_H_44_O_7_NP	452.2773			0.32	391, 255, 214, 196, 153	+	+
**33**	l-PC (16:0)	17.77^a^	C_24_H_50_O_7_NP		540.3288		-1.47	480, 391, 255, 242, 224	+	+
**34**	9,10-DHSA	17.98^b^	C_18_H_36_O_4_	315.2536			2.04	297,279, 201, 187, 171	+	+
**35**	DGMG (18:1)	18.24^b^	C_33_H_60_O_14_	679.3911^b^	725.3958		1.18^b^	415, 397, 323, 281, 235	+	+
**36**	l-PE (18:1)	18.39^b^	C_23_H_46_O_7_NP	478.2933			0.95	417, 281, 214, 196, 153	+	+
**37**	l-PC (18:1)	18.50^a^	C_26_H_52_O_7_NP		566.3454^a^	522.3538^d^	0.27^a^	506, 417, 281 242, 224	+	+
**38**	l-PE (18:1)	19.36^b^	C_23_H_46_O_7_NP	478.2935			1.47	417, 281, 214, 196, 153	+	+
**39**	l-PC (18:1)	19.53^a^	C_26_H_52_O_7_NP		566.3458^a^	522.3538^d^	1.03^a^	506, 417, 281, 242, 224	+	+
**40**	9-HODE (10, 12)	22.28^b^	C_18_H_32_O_3_	295.2271			1.35	277, 171	+	+
**41**	acyldiglycoside (18:1)	21.65^b^	C_30_H_54_O_12_	605.3537^b^	651.3589		0.55^b^	341, 323, 281	+	+
**42**	acyldiglycoside (18:1)	22.08^b^	C_30_H_54_O_12_	605.3532^b^	651.3590		0.06^b^	341, 323, 281	+	+
**43**	l-PS (18:2)	22.30^b^	C_24_H_44_O_9_NP	520.2676			0.89	502, 433, 415, 279, 153	+	+
**44**	acyldiglycoside (18:1)	22.88^b^	C_30_H_54_O_12_	605.3533^b^	651.3589		0.12^b^	341, 323, 281	–	+
**45**	l-PI (16:1)	23.55^b^	C_25_H_47_O_12_P	569.2725			0.56	389, 315, 253, 241, 223	–	+
**46**	DGMG (18:0)	23.93^b^	C_33_H_62_O_14_	681.4067^b^	727.4115		1.09^b^	415, 397, 323, 235	–	+
**47**	l-PE (18:0)	23.94^b^	C_23_H_48_O_7_NP	480.3089			0.97	419, 283, 214, 196, 153	+	+
**48**	l-PI (16:1)	24.32^b^	C_25_H_47_O_12_P	569.2721			-0.09	389, 315, 253, 241, 223	–	+
**49**	acyldiglycoside (18:1)	24.45^b^	C_30_H_54_O_12_	605.3544^b^	651.3594		1.22^b^	281	+	+
**50**	l-PC (18:0)	25.24^b^	C_26_H_54_O_7_NP		568.3611		0.45	508, 283, 242, 224	+	+
**51**	l-PI (18:2)	25.57^b^	C_27_H_49_O_12_P	595.2880			0.29	415, 333, 315, 279, 241, 223, 171	+	+
**52**	l-PE (18:0)	25.62^b^	C_23_H_48_O_7_NP	480.3089			0.97	419, 283, 214, 196, 153	–	+
**53**	l-PS (18:1)	26.10^b^	C_24_H_46_O_9_NP	522.2822			-0.91	504, 435, 417, 283, 153	+	+
**54**	l-PC (18:0)	27.04^b^	C_26_H_54_O_7_NP		568.3612		0.55	508, 419, 283, 242, 224	+	+
**55**	l-PI (18:2)	27.61^b^	C_27_H_49_O_12_P	595.2882			0.71	415, 333, 315, 279, 241, 223, 171	+	+
**56**	NA-GPE (18:2)	27.71^b^	C_23_H_44_O_7_NP	476.2772^b^			0.05^b^	402, 384, 278, 214, 171, 153	+	+
					478.2944^d^	3.25^d^	460, 324, 306, 280	+	+
**57**	l-PS (18:1)	29.13^b^	C_24_H_46_O_9_NP	522.2830			0.60	504, 435, 417, 281, 153	–	+
**58**	l-PI (16:0)	29.34^b^	C_25_H_49_O_12_P	571.2876			-0.33	409, 391, 333, 315, 255, 241, 223, 171	+	+
**59**	l-PI (16:0)	32.22^b^	C_25_H_49_O_12_P	571.2880			0.40	409, 391, 333, 315, 255, 241, 223, 171	+	+
**60**	SQMG (18:2)	33.45^b^	C_27_H_48_O_11_S	579.2839			0.88	317, 299, 279, 225, 207	+	+
**61**	l-PI (18:1)	34.29^b^	C_27_H_51_O_12_P	597.3049			2.39	435, 417, 333, 315, 281, 241, 223, 171	+	+
**62**	SQMG (18:2)	35.28^b^	C_27_H_48_O_11_S	579.2837			0.67	317, 299, 279, 225, 207	+	+
**63**	l-PI (18:1)	37.21^b^	C_27_H_51_O_12_P	597.3043			1.47	435, 417, 333, 315, 281, 241, 223, 171	+	+
**64**	NA-GPE (18:1)	37.25^b^	C_23_H_46_O_7_NP	478.2934^b^			1.28^b^	404, 386, 280, 214, 171, 153	+	+
					480.3085^d^	0.07^d^	462, 326, 308, 282	+	+
**65**	SQMG (16:0)	39.43^b^	C_25_H_48_O_11_S	555.2835			0.27	317, 299, 255, 225, 207	+	+
**66**	SQMG (16:0)	42.29^b^	C_25_H_48_O_11_S	555.2838			0.81	317, 299, 255, 225, 207	+	+
**67**	MAG (16:0)	46.56^c^	C_19_H_38_O_4_			331.2835	-2.25	313, 281, 257, 239	+	+
**68**	MAG (18:1)	48.99^c^	C_21_H_40_O_4_			357.2987	-3.49	339, 283, 265	+	+
**69**	SQMG (18:1)	50.51^b^	C_27_H_50_O_11_S	581.2994			0.64	317, 299, 281, 225, 207	+	+
**70**	MAG (18:0)	54.35^c^	C_21_H_42_O_4_			359.3145	-2.99	341, 285, 267, 239	+	+
**71**	MAG (18:0)	55.59^c^	C_21_H_42_O_4_			359.3147	-2.47	341, 285, 267, 239	+	+

^a^derived by LC-HRMS analysis of NPR on Atlantis T3 column in negative ion mode; ^b^derived by LC-HRMS analysis of NPF on Atlantis T3 column in negative ion mode, ^c^derived by LC-HRMS analysis of NPR on Atlantis T3 column in positive ion mode, ^d^derived by -MS analysis of NPF on Atlantis T3 column in positive ion mode.

The 18-carbon hydroxylated acyl chain is abbreviated as 18:2-1O to indicate two double bond equivalent and one oxygen atom beyond the carbonyl group.

*Compounds characterized by NMR.

+: metabolites present in the extract; -: metabolites absent in the extract.

**Table 2:** Polar lipids putatively identified in fresh and roasted “Nocciola Piemonte” extracts by using a RP-C4 column for the chromatographic separation.

n°	Compound	*R_t_* (min)	Molecular Formula	[M-H]^-^	[(M+FA)-H]^-^	Error (ppm)	Characteristic product ions	NPR	NPF
**72**	l-PG (16:0)	12.57^b^	C_22_H_45_O_9_P	483.2722		-1.29	391, 255, 245, 227, 153	–	+
**73**	l-PA (18:2)	12.88^b^	C_21_H_39_O_7_P	433.2349		-0.11	415, 279, 153, 135	+	+
**74**	l-PG (16:0)	12.93^b^	C_22_H_45_O_9_P	483.2722		0.86	391, 255, 245, 227, 153	+	+
**75**	l-PG (18:1)	13.29^b^	C_24_H_47_O_9_P	509.2874		-0.03	417, 281, 245, 227, 153	+	+
**76**	l-PG (18:1)	13.54^b^	C_24_H_47_O_9_P	509.2870		-0.86	417, 281, 245, 227, 153	+	+
**77**	l-PA (18:2)	13.59^b^	C_21_H_39_O_7_P	433.2350		0.12	415, 279, 153, 135	+	+
**78**	l-PA (18:1)	13.75^b^	C_21_H_41_O_7_P	435.2500		-1.42	417, 281, 153, 135	–	+
**79**	l-PA (16:0)	14.06^b^	C_19_H_39_O_7_P	409.2359		2.21	391, 255, 153	–	+
**80**	l-PA (18:1)	14.67^b^	C_21_H_41_O_7_P	435.2504		-0.43	417, 281, 153, 135	–	+
**81**	PI (18:2-1O;16:0)	19.78^a^	C_43_H_79_O_14_P	849.5112		-1.34	831, 687, 593, 553, 431, 391, 315, 295, 255, 241	+	**-**
**82**	PI (18:2-1O;18:1)	20.50^a^	C_45_H_81_O_14_P	875.5273		-0.84	857, 713, 611, 593, 579, 431, 417, 315, 295, 281	+	–
**83**	PI (18:1;18:2-1O)	22.29^a^	C_45_H_81_O_14_P	875.5299		2.16	713, 611, 593, 579, 431, 417, 315, 297, 295, 281	+	–
**84**	PI (18:2;16:1)	23.67^a^	C_43_H_77_O_13_P	831.5022		0.49^a^	577, 569, 551, 389, 297, 279, 253, 241	+	+
**85**	PI (18:3; 16:0)	23.73^a^	C_43_H_77_O_13_P	831.5030		1.46	575, 389, 277, 255, 241	+	+
**86**	PI (18:2;18:2)	24.34^a^	C_45_H_79_O_13_P	857.5177		0.26	595, 577, 433, 415, 315, 297, 279, 241	+	+
**87**	GlcCer (d18:2; h16:0)	24.76^a^	C_40_H_75_O_9_N	712.5353		-0.74	550, 532, 271, 225; MS^3^(550): 532, 314, 312, 296, 271, 270, 253, 235, 225	+	+
**88**	PI (16:0;18:2)	25.48^a^	C_43_H_79_O_13_P	833.5174		-0.09	595, 577, 571, 553, 415, 391, 315, 297, 279, 255, 241	+	+
**89**	PI (18:1;18:2)	26.63^a^	C_45_H_81_O_13_P	859.5334		0.35	597, 595, 579, 577, 435, 417, 315, 297, 281, 279, 241	+	+
**90**	PI (16:0;18:1)	28.08^a^	C_43_H_81_O_13_P	835.5334		0.41	673, 579, 571, 553, 417, 391, 315, 297, 281, 255, 241	+	+
**91**	GlcCer (d18:2; h18:0)	28.86^a^	C_42_H_79_O_9_N	740.5677		0.86	578, 560; MS^3^(578): 560, 530, 342, 340, 324, 299, 298, 281, 253	+	+
**92**	PI (18:1;18:1)	29.48^a^	C_45_H_83_O_13_P	861.5492		0.53	699, 597, 579, 435, 417, 315, 297, 281, 241	+	+
**93**	PE (18:2; 18:2)	29.85^a^	C_41_H_74_O_8_NP	738.5078		1.36	476, 458, 279	+	+
**94**	SQDG (16:0; 18:1)	30.83^a^	C_43_H_80_O_12_S	819.5277		-1.18	563, 555, 537, 281, 255; MS^3^(563): 545, 519, 299, 225	+	+
**95**	SQDG (18:1; 18:1)	32.33^a^	C_45_H_82_O_12_S	845.5431		-1.46	581, 563, 281; MS^3^(563): 299, 225	+	+
**96**	PG (16:0;18:2)	31.35^a^	C_40_H_75_O_10_P	745.5005		-1.15	671, 507, 489, 483, 465, 415, 391, 279, 255, 227	+	+
**97**	PE (16:0; 18:2)	31.81^a^	C_39_H_74_O_8_NP	714.5075		0,92	476, 458, 452, 434, 279, 255	+	+
**98**	PG (18:1;18:2)	32.80^a^	C_42_H_77_O_10_P	771.5182		1.42	697, 509, 507, 491, 489, 417, 415, 281, 279, 245	+	+
**99**	PI (18:0;18:1)	32.90^a^	C_45_H_85_O_13_P	863.5647		0,36	599, 581, 437, 419, 315, 297, 283, 281, 241	+	+
**100**	PE (18:2; 18:1)	33.11^a^	C_41_H_76_O_8_NP	740.5232		0,99	478, 476, 460, 458, 281, 279	+	+
**101**	PC (18:2;18:2)	34.24^a^	C_44_H_80_O_8_NP		826.5597	0.52	766, 504, 279	+	+
**102**	PG (16:0;18:1)	34.76^a^	C_40_H_77_O_10_P	747.5176		0.69	673, 509, 491, 483, 465, 417, 391, 281, 255, 245, 227	+	+
**103**	PE (16:0; 18:1)	35.18^a^	C_39_H_76_O_8_NP	716.5234		1,25	452, 434, 391, 281, 255	+	+
**104**	SQDG (18:0; 18:1)	35.95^a^	C_45_H_84_O_12_S	847.5591		-0.98	565, 563, 283, 281; MS^3^(563): 225	+	+
**105**	PG (18:1;18:1)	36.31^a^	C_42_H_79_O_10_P	773.5339		1.50	699, 509, 491, 435, 417, 281, 227	+	+
**106**	PC (16:0;18:2)	36.57^a^	C_42_H_80_O_8_NP		802.5600	0.92	742, 504, 480, 279, 255	+	+
**107**	PE (18:1; 18:1)	36.68^a^	C_41_H_78_O_8_NP	742.5391		1,33	486, 478, 460, 281	+	+
**108**	PA (18:2;18:2)	37.96^b^	C_39_H_69_O_8_P	695.4647		0.13	433, 415, 279	–	+
**109**	PC (18:2;18:1)	37.98^a^	C_44_H_82_O_8_NP		828.5746	-0.39	768, 506, 504, 488, 486, 281, 279	+	+
**110**	PA (18:1;18:2)	40.45^b^	C_39_H_71_O_8_P	697.4814		1.57	435, 433, 417, 415, 281, 279	+	+
**111**	PA (16:0;18:2)	40.82^b^	C_37_H_69_O_8_P	671.4647		0.04	433, 415, 409, 391, 279, 255	**-**	+
**112**	PC (16:0;18:1)	40.99^a^	C_42_H_82_O_8_NP		804.5750	0.12	744, 488, 480, 462, 281, 255	+	+
**113**	PC (18:1;18:1)	42.55^a^	C_44_H_84_O_8_NP		830.5911	0.60	770, 506, 488, 281	+	+
**114**	PA (16:0;18:1)	45.71^b^	C_37_H_71_O_8_P	673.4810		1.01	435, 417, 409, 391, 281, 255	+	+
**115**	PA (18:1;18:1)	46.84^b^	C_39_H_73_O_8_P	699.4966		1.01	435, 417, 281	+	+
**116**	PA (18:1;20:2)	49.31^b^	C_41_H_75_O_8_P	725.5107		-0.84	461, 443, 417, 307, 281	–	+
**117**	PC (18:1;18:0)	49.59^a^	C_44_H_86_O_8_NP		832.6050	-1.41	772, 508, 283	+	+
**118**	PA (18:1;18:0)	50.82^b^	C_39_H_75_O_8_P	701.5110		-0.59	437, 419, 417, 283, 281	–	+
**119**	PA (18:1;20:1)	52.42^b^	C_41_H_77_O_8_P	727.5267		-0.56	463, 445, 417, 309, 281	–	+

^a^derived by LC-HRMS analysis of NPR on Symmetry C4 column in negative ion mode; ^b^derived by LC-HRMS analysis of NPF on Symmetry C4 column in negative ion mode.

In GlcCer nomenclature, the 2-hydroxylated fatty acids are indicated by the letter “h” added to their lipid number (i.e., h16:0).

+: metabolites present in the extract; -: metabolites absent in the extract. The 18-carbon hydroxylated acyl chain is abbreviated as 18:2-1O to indicate two double bond equivalent and one oxygen atom beyond the carbonyl group.

### Oxylipins identification

The analysis of LC-(-)ESI/HRMS profiles allowed to detect both in NPF and in NPR extracts the compounds **12**, **19**, **23**, **29**, **34**, and **40** (**Table 1**) ascribable to oxylipins, which are hydroxyl fatty acids having different unsaturation degree and number of hydroxyl groups, and mainly deriving from the oxidative metabolism of essential PUFA, such as α-linolenic acid (ALA) (18:3-3) and linoleic acid (LA) (18:2-6) (Masullo et al., 2021a). The analysis of their tandem mass spectra highlighted the occurrence of characteristic product ions and diagnostic neutral losses generated by molecular rearrangements involving the head and the end of the acyl chain that, according to literature data, allowed to putatively assign the position on the fatty acyl chain of both hydroxyl groups and double bonds (Richardson et al., 2017; Napolitano et al., 2018). By this way, the finding of the diagnostic product ion at *m/z* 171 (originated by the rearrangement to aldehyde of the hydroxyl function at C9, that caused the subsequent cleavage of the C9–C10 bond and the consequent shortening of the acyl chain, retaining the head-carboxyl group as COO^-^), allowed the structural assignment of oxylipins **12**, **19**, **23**, **29**, **34**, and **40** (**Table 1**) (Levandi et al., 2009). Analogously, the detection in the tandem mass spectrum of the diagnostic peak at *m/z* 183 (corresponding to a molecule of hydroxylated heptanal deriving from the end-part of the acyl chain by breakdown of the C11 and C12 bond, subsequent to the typical CHOH→CHO rearrangement involving the hydroxyl group at C12 guided the identification of oxylipin 19 (Napolitano et al., 2018).

### Phospholipids and N-acylglycerophosphatidylethanolamines identification

The analysis of mass spectrometric data of both NPF and NPR extracts allowed the identification of several compounds ascribable to different classes of phospholipids and their lyso-forms, in which only one of the *sn-1*/*sn-2* positions of the glycerol unit was fatty acylated.

In MS/MS experiments, the different classes of phospholipids are characterized by the production of typical class-featured fragmentation patterns, promptly allowing to distinguish them. By this way, the occurrence of several compounds identifiable as lyso-phosphatidylinositols (l-PI) (45, 48, 51, 55, 58, 59, 61, and 63) and phosphatidylinositols (PI) (81–86, 88–90, 92>, and 99) (**Tables 1**, **2**) could be ascertained by the occurrence in their tandem mass spectrum of the diagnostic product ion at *m/z* 241, corresponding to the mono-dehydrated form of the inositol hydrophilic head residue linked to the phosphate group. Peaks originated by neutral loss of the polar head group alone, the [(M-162)-H]^−^ and [(M-180)-H]^−^ ions, respectively) (**Tables 1**, **2**) could be observed. The product ions yielded from each [M-H]^−^ on by neutral loss of one (in l-PI case) or two fatty acyl moieties (in PI case), along with the R_x_COO^−^ carboxylate anions allowed to assign the nature of the fatty acids and, in the case of PI, their regiospecificity, by considering that the fatty acid removed from the *sn-1* position yielded a more intense R_1_COO^−^ anion than that removed from the *sn-2* position (**Tables 1**, **2**) (Geng et al., 2015).

The analysis of the MS data of **21**, **25**, **27**, **32**, **36**, **38**, **47**, and **52** allowed to assign these compounds to the lyso-phosphatidylethanolamine (l-PE) class on the basis both of their molecular formula, containing a common NO_7_P heteroatom composition, and of the presence of diagnostic product ions at *m/z* 214 and 196, composed of the head group of this lipid class and the glycerophosphatidyl unit, as whole or in the mono-dehydrated form (**Table 1**). The nature of the fatty acid could be inferred from the observation of a main R_x_COO^−^ product ion (**Table 1**) (Geng et al., 2015). The MS^2^ spectra of PE species (**93**, **97**, **100**, **103**, and **107**) were instead characterized by the occurrence of minor [(M-R_x_COOH)-H]^−^ and [(M-R_x_=CO)-H] ^−^ product ions along with abundant carboxylate anions originated by neutral loss of 197 Da (corresponding to the mono-dehydrated form of glycero-phosphatidylamine) from the [(M-R_x_=CO)-H]^−^ ion (**Table 2**). Their acylation position on the glycerol unit could be deduced from their relative intensity ratio with the fatty acid at *sn-2* position generating a more intense anion than that at *sn-1* position (R_2_COO^−^ > R_1_COO^−^) (Napolitano et al., 2018).

Compounds **56** and **64** (**Table 1**), occurring both in fresh and roasted kernels, were identified as N-acylglycerophosphatidylethanolamines (NA-GPE) molecules (Klockmann et al., 2016; Napolitano et al., 2018), that is, lipid compounds in which the fatty acid is involved in an amide linkage with the amino head group of glycerophosphoethanolamine (Tsuboi et al., 2011).

The analysis of mass spectrometric data acquired in negative ion mode allowed to identify, both in NPR and NPF extracts, a good number of metabolites (**13**, **14**, **17**–**18**, **22**, **26**, **30**, **33**, **37**, **39**, **50**, **54**, **101**, **106**, **109**, **112**–**113**, and **117**) belonging to another class of phospholipids, characterized by a heteroatom composition including an atom of nitrogen and another of phosphorous and by an MS/MS spectrum displaying a main peak originated by neutral loss of 60 Da from the pseudomolecular anion (**Tables 1**, **2**,his product ion represents the typical anion formed from PC derivatives when subjected to CID under negative ionization conditions. In the (^−^)ESI mode the pseudomolecular anion of this phospholipid species is obtained as adduct with formic acid, that, in MS/MS experiment, is promptly removed as methyl formate (60 Da), generating the diagnostic [M-15]^−^ ion from which any other product ion derives (**Tables 1**, **2**) (Geng et al., 2015; Napolitano et al., 2018). The occurrence in the (-)ESI/MSMS spectrum of the product ion obtained by neutral loss of a dimethylaminoethanol unit (89 Da) from the [M-15]^−^ ion confirmed this assignment, along with the occurrence of two minor product ions, at *m/z* 241 and 224, corresponding to the glycerol-phosphatidyldimethylethanolamine as whole or mono-dehydrated anion, respectively (**Table 1**).

Moreover, the analysis of the mass spectra acquired for chromatographic peaks yielded by NPF and NPR investigation on RP-C18 column allowed to ascertain the occurrence in both hazelnut extracts of some lyso-phosphatidylserines (l-PS) (**43**, **53**, and **57**) by observing the relative tandem mass spectra characterized by major product ions formed by neutral loss of 105 Da and 87 Da, corresponding to the serine headgroup removed as whole or mono-dehydrated unit (**Table 1**).

The occurrence of lyso-phosphatidylglycerol (l-PG) (**72** and **74**–**76**) and PG (**96**, **98**, **102**, and **105**) species both in NPR and NPF extracts could be ascertained by analyzing in the tandem mass spectra diagnostic product ions at *m/z* 245 and 227, corresponding to the whole and mono-dehydrated glycerophosphoglycerol anions, respectively, along with the product ion originated by neutral loss of 92 Da corresponding to a glycerol unit (**Table 2**) (Cerulli et al., 2021).

Finally, the occurrence in both NPR and NPF of phospholipids belonging to the lyso-phosphatidic acid (l-PA)/PA class (**Table 2**) could be highlighted by the finding of a main peak at *m/z* 153 in MSMS spectra of l-PA (**73** and **77**–**80**) and of a main peak obtained by neutral loss of 154 Da from the [(M-H)-R_x_C=O]^−^ anion in MSMS spectra of PA (**108**, **110**–**111**, **114**–**116**, and **118**–**119**).

### Glycolipids, monoacylglycerols, and sphingolipids identification

The analysis of MS data obtained by using the RP-C18 column for the chromatographic separation allowed to putatively identify in both NPR and NPF extracts digalactosylmonoacylglycerol (DGMG) species (**15**–**16**, **20**, **24**, **28**, **31**, **35**, and **46**), differing for the unsaturation degree and/or regiospecificity of the acyl group (**Table 1**). Particularly diagnostic for the class-assignment were the product ions occurring in the tandem mass spectrum at *m/z* 415 and 397, corresponding to the digalactosylglycerol as whole or as mono-dehydrated anion, respectively, and at *m/z* 235, relative to the mono-dehydrated galactosylglycerol anion (**Table 1**) (Napolitano et al., 2018; D’Urso et al., 2020).

Sulfoquinovosylmonoacylglycerols (SQMGs) (**60**, **62**, **65**–**66**, and **69**) could be identified by the occurrence of the typical product ions at *m/z* 299 and 225, referable to the mono-dehydrated glycerosulfoquinovose anion and to the sulphured sugar anion rearranged after removal from the glycerol unit (**Table 1**) (Zianni et al., 2013; Fu et al., 2018). In both fresh and roasted hazelnut extracts, by using the RP-C4 column, sulfoquinovosylglycerols in which both *sn*-1 and *sn*-2 glycerol positions were acylated (SQDG) (**94**–**95** and **104**) (**Table 2**) were detectable. For these compounds the regiochemical characterization could be established on the basis of the intensities of the [(M-R_x_COOH)-H]^−^ anions produced in MS/MS spectra according to literature reports (Zianni et al., 2013; Napolitano et al., 2018)

Derivatives of octadecenoic acids esterified with two glycosyl units (**41**–**42**, **44**, and **49**) were detectable in LC-(-)ESI/HRMS profiles acquired for both NPR and NPF extracts by using the RP-C18 column (**Table 1**). In particular, the tandem mass spectrum of these acyldiglycosides was characterized by the occurrence of product ions at *m/z* 341 and 323, corresponding to the diglycosyl anion as whole or in mono-dehydrated form (Rahman and Akhtar, 2016; Sultana et al., 2018).

In agreement with literature data, both molecular formula and fragmentation pattern of peaks **67**–**68**, **70**, and **71** concurred to define them as MAG. Their tandem mass spectrum, acquired in positive ion mode, was typically characterized by the occurrence of product ions formed by neutral loss of 92 and 74 Da, and corresponding to [*R* = CO+H]^+^ and [RCOOH+H]^+^ cations, respectively (**Table 1**) (Della Corte et al., 2015; Napolitano et al., 2018).

Finally, the LC-(-)ESI/HRMS profiles obtained by using the RP-C4 column highlighted the occurrence in both “Nocciola Piemonte” extracts of two peaks (87 and 91) yielding MS^2^ spectra characterized by abundant [(M-162)-H]^−^ and [(M-180)-H]^−^ ions (**Table 2**) corresponding to ceramides (**Table 1**). These compounds are lipids structurally made up of a long-chain base, in plant usually consisting of an 18-carbon atom backbone supporting hydroxyl groups at C1 and C3 and an amine group at C2, linked by *N*-acylation to a fatty acid generally made up of 14–26 carbon atoms and usually hydroxylated at C-2. The comparison with literature data permitted to identify compounds **87** and **91** as glycosylceramides (GlcCers) (Alasalvar and Pelvan, 2011).

### Compositional distribution of polar lipids

The knowledge of the mechanism of lipids fragmentation was used to design an analytical strategy that, by monitoring the characteristic transitions for each lipid class in MRM experiments, allowed to obtain a quantitative distribution, in relative terms, of the main polar lipid families occurring in both NPR and NPF, that is, phosho- and glycolipids.

**Figure 1:**
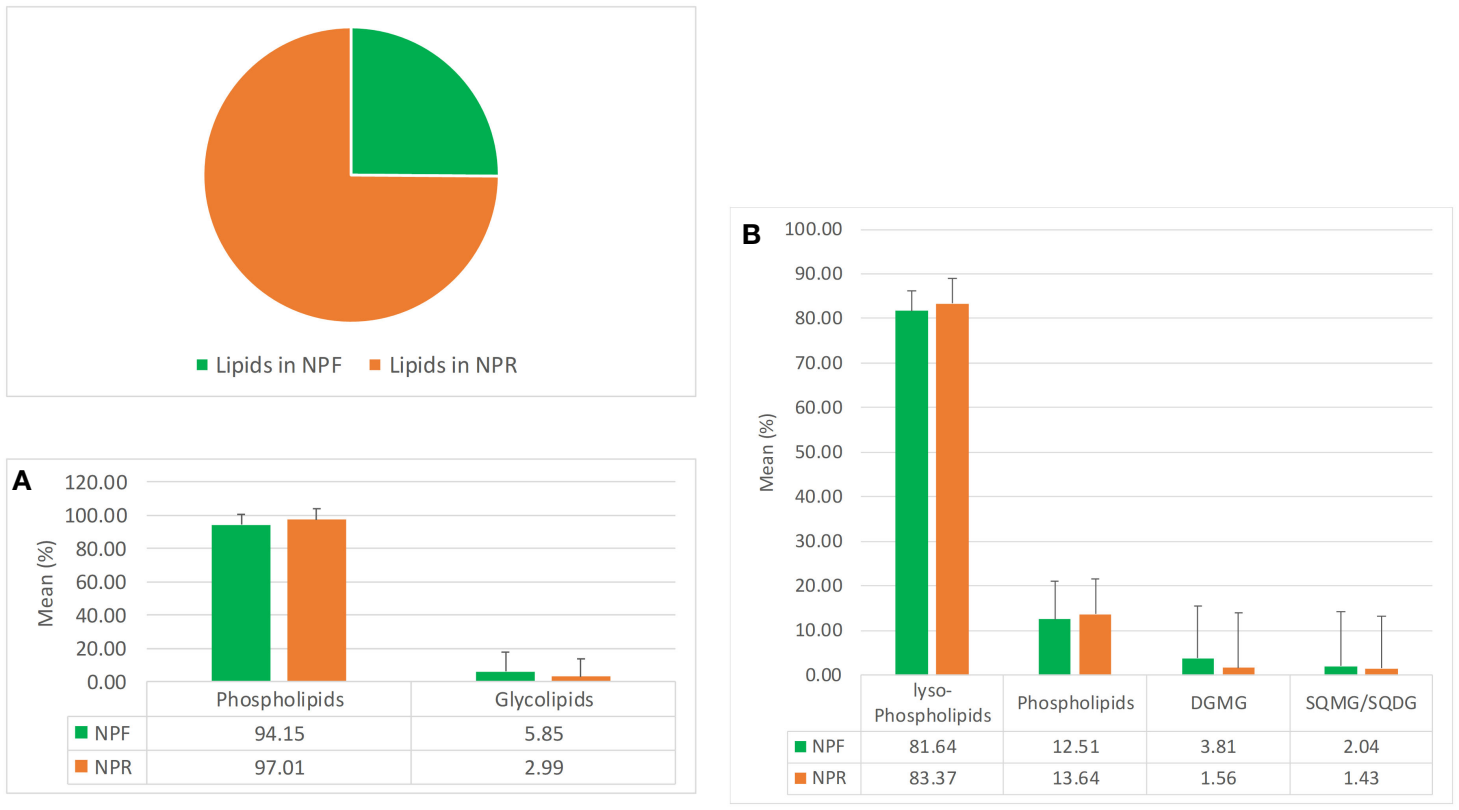
Mean quantitative responses and relative standard deviation, expressed as percentage, of total lipids (pie chart), **(A)** phosho- and glycolipids families, and **(B)** phosho- and glycolipids classes occurring in NPR and NPF.

The pie chart in **Figure 1** clearly indicates that NPR was about threefolds richer in lipids than NPF, which could be explained by a better extractability following the destruction of hazelnut tissues by roasting, in agreement with previous works carried out on different matrices such as safflower, rice germ, argan, pumpkin, and mustard seeds (Belcadi-Haloui et al., 2018). The bar histograms shown in **Figure 1A** indicate that the relationship existing between the two main lipid families present in NPR and NPF was basically the same, highlighting in both roasted and fresh kernels the clear prevalence in relative terms of the phospholipids family versus that of glycolipids, with the latter being even less represented in NPR with respect to the first one. In particular, both in NPR and in NPF the lyso-phospholipid class resulted to be the most represented lipid class in relative terms, followed by those of phospholipids, digalactosylmonoacylglicerols, and sulfoquinovosyl species (**Figure 1B**).

**Figure 2:**
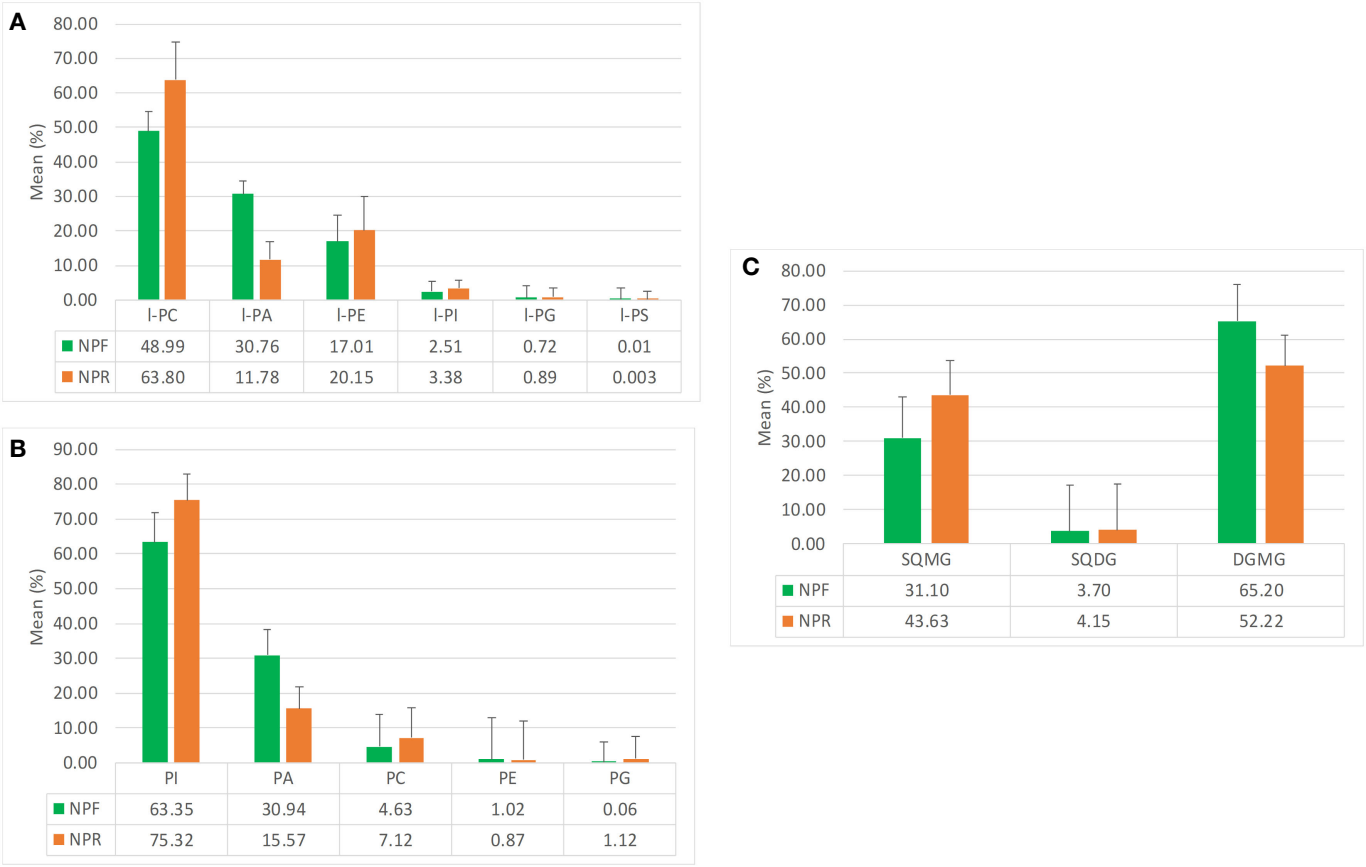
Mean quantitative responses and relative standard deviation, expressed as percentage, of **(A)** lyso-phospholipids classes, **(B)** phosholipids classes, and **(C)** glycolipids classes identified in NPR and NPF.

The analysis of the quantitative distribution in relative terms of the single classes of lyso-phospholipids allowed to affirm that in both NPF and NPR extracts l-PC was the most represented lyso-phospholipid class, followed in the order by l-PA and l-PE, l-PI, l-PG, and l-PS (**Figure 2A**).

Notwithstanding this, some peculiar differences could be noted between the two extracts. In particular, the kernel roasting seemed to have a positive effect on the level of l-PC, l-PE, l-PI, and l-PG, that showed an increase in relative terms (more consistent and significative for the first than for the other three classes) with respect to the same classes in NPF, and a contemporary negative effect on the level of l-PA and l-PS, characterized by an evident decrease with respect to the NPF (**Figure 2A**). It is noteworthy that in NPR the l-PC class was fivefold and 21-fold more represented than l-PA and l-PS, respectively, while the relationship of l-PC with the other lyso-phospholipid classes remained substantially unvaried with respect to that observed between the same classes in NPF. Differing from the distribution trend observed for lyso-phospholipids, among the considered phospholipid classes, PI resulted the most represented, both in NPF and in NPR, followed by PA, PC, PE, and PG (**Figure 2B**). Once again, the roasting process seemed to have effects on phospholipid relative quantitative profile yielding to, analogously to what observed in the case of lyso-phospholipids, an increase of the level of PI, PC, and PG, and a decrease of PA and PE, much more relevant for the first than for the second (**Figure 2B**). So, our results led us to consider that probably the used roasting conditions could have determined the destruction of membrane structures of hazelnut oil bodies causing the release of phospholipids such as l-PI, l-PC, l-PG, PI, PC, and PG leading to their increase in the NPR extract, and the contemporary thermal degradation of more susceptible phospholipids such as l-PA, l-PS, PAs, and PEs, in agreement with literature data available for matrices other than hazelnut (Kim et al., 2002; Zhang et al., 2021; Zhang et al., 2022).

Finally, by considering the glycolipid family, both in NPF and in NPR the DGMG resulted to be the most represented glycolipid class in relative terms, with the SQMG class being the most represented between the two considered sulfoquinovosyl classes (**Figure 2C**). In this case, the analysis of the relationships existing between the different glycolipid classes in NPR highlighted an evident roasting effect determining the decrease of the DGMG level in favor of the increase of the level of both SGMG and SQDG (**Figure 2C**).

### Phenolic derivatives

The analysis of the LC-(-)ESI/HRMS profile obtained by using the RP-C18 column highlighted some peaks that, on the basis of their molecular formula and fragmentation pattern, could be identified as mono- and diglycosylated flavonoids (**Table 1**). In particular, tandem mass spectra of compounds **6**, **7**, and **9** allowed to promptly identify them as mono-glycosylated flavonoids showing as aglycone myricetin, quercetin, and kaempferol, respectively (**Table 1**). The analysis of MSMS spectrum of compounds **3** and **5** allowed instead to define them as diglycosylated flavonols, showing as main peak the aglycone ions at *m/z* 301 and *m/z* 285 corresponding to quercetin and kaempferol, respectively, and as minor product ions those originated from the [M-H]^−^ ion by neutral loss of one mono-dehydrated hexose unit and from the [(M-H)-162]^−^ ion by neutral loss of 120 Da via internal breakdown of the second hexose unit (**Table 1**). In agreement with literature data, this fragmentation pattern suggested that the 3-*O* position of the aglycone core had to be involved in the glycosylation with the two hexose units, that in turn had to be each other linked via a (1→2)-interglycosidic linkage (Masullo et al., 2016; Cerulli et al., 2020; Loizzo et al., 2021). Noteworthy, compounds **3**, **7**, and **9** could be detected in both NPR and NPF extracts, while compounds **5** and **6** were evident only in NPR extract.

Moreover, the analysis of mass spectrometric data acquired by carrying out the chromatographic separation on RP-C18 column allowed to ascertain the occurrence of four phenolic compounds (**4**, **8**, **10**, and **11**) ascribable to diarylheptanoid derivatives (**Table 1**) (Masullo et al., 2017; Cerulli et al., 2018c). In particular, they were more evident in NPR than in NPF extract, which showed only compound 10.

### Isolation and NMR structural elucidation of phenolic derivatives

**Figure 3:**
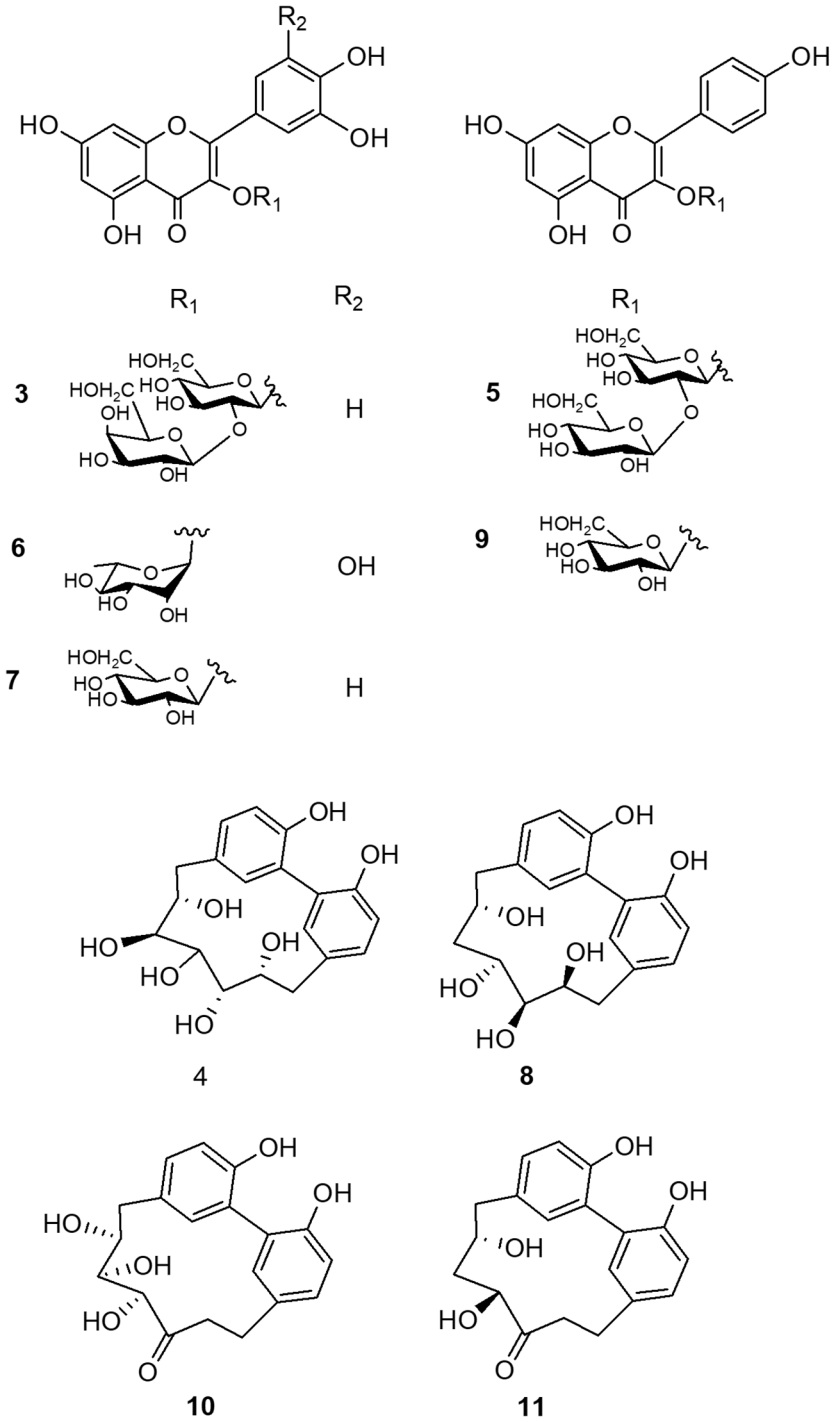
Compounds isolated from “Nocciola Piemonte” roasted hazelnut.

With the aim to complete and unequivocally characterize the molecular structure of flavonoid and diarylheptanoids derivatives detected in LC-ESI/HRMS profile, the NPR *n*-butanol extract was fractionated on Sephadex LH-20 and the obtained fractions were purified by RP-RI/HPLC. The comparison of NMR data acquired for isolated compounds with those reported in literature allowed to identify the diarylheptanoids as: giffonin P (**4**), giffonin L (**8**), carpinontriol B (**10**), and giffonin V (**11**), and the flavonoids as: quercetin 3-*O*-β-D-galactopyranosyl-(**1**→**2**)-β-D-glucopyranoside (**3**), kaempferol 3-*O*-β-D-glucopyranosyl-(**1**→**2**)-β-D-glucopyranoside (**5**), myricetin-3-*O*-α-L-rhamnopyranoside (**6**), quercetin 3-*O*-β-D-glucopyranoside (**7**), and kaempferol-3-*O*-β-D-glucopyranoside (**9**) (**Figure 3** and **Table 1**) (Masullo et al., 2015a; Masullo et al., 2016; Cerulli et al., 2017).

### TEAC assay of “Nocciola Piemonte” extracts and phenolic compounds

TEAC assay was used to evaluate the antioxidant ability of isolated specialized metabolites (**3**–**11**). TEAC value is expressed as the concentration of Trolox solution with antioxidant potential equivalent to a 1 mM concentration of the test sample. Among the tested compounds, flavonoid glycosides showed a good antioxidant activity; in particular, compounds **3** and **6** displayed a radical-scavenging activity similar to that of quercetin (reference compound) (TEAC value 2.03 mM). The diarylheptanoids (**4**, **8**, **10**, and **11**), characterized by the presence at least of two phenolic groups, showed a moderate radical-scavenging capacity (**Table 3**).

**Table 3:** Free radical scavenging activities by TEAC assay and inhibitory effects on plasma lipid peroxidation induced by H_2_O_2_ or H_2_O_2_/Fe^2+^ measured for compounds **3**−**11**.

compound	TEAC value (mM)	Inhibition of lipid peroxidation induced by H_2_O_2_ (%)	Inhibition of lipid peroxidation induced by H_2_O_2_/Fe^2+^ (%)
**3**	2.00 ± 0.03	19.9 ± 2.6	18.8 ± 3.1
**4**	0.99 ± 0.01	33.6 ± 3.2	11.4 ± 2.2
**5**	1.65 ± 0.02	35.4 ± 3.6*	14.2 ± 2.1
**6**	2.06 ± 0.03	44.4 ± 4.2	34.1 ± 3.4
**7**	1.98 ± 0.03	23.1 ± 2.8	19.9 ± 2.0
**8**	0.75 ± 0.01	33.3 ± 3.3	14.4 ± 1.9
**9**	1.55 ± 0.02	5.7 ± 1.6	20.1 ± 3.6
**10**	0.87 ± 0.01	32.0 ± 3.4	24.4 ± 3.3
**11**	0.60 ± 0.01	–	7.0 ± 2.3
**quercetin^a^**	2.03 ± 0.03	–	–
**curcumin^b^**		20.6 ± 4.1	23.2 ± 2.4

^a^reference compound used for TEAC assay; ^b^reference compound used for TBARS assay. *p < 0.01. -, Quercetin was used as reference standard only for TEAC assay.

### Biological evaluation of phenolic compounds in TBARS assay

On the basis of the antioxidant activity reported for phenolic compounds and more specifically for giffonins isolated from *C. avellana* byproducts, the phenolic compounds isolated from the extract of roasted “Nocciola Piemonte” were evaluated by measuring the concentration of TBARS (Kolodziejczyk et al., 2009; Masullo et al., 2015b; Masullo et al., 2016; Cerulli et al., 2017).

This assay highlighted that the tested compounds protected plasma against lipid peroxidation induced by H_2_O_2_ and H_2_O_2_/Fe^2+^. In detail, phenolic compounds and curcumin (reference compound) were assayed at different concentrations (0.1 µM–100 µM; 30 min, at 37°C). All tested compounds and reference compound did not exhibit effect on auto-peroxidation of human plasma (data not shown).

Most of the tested compounds at concentration of 10 µM were able to reduce H_2_O_2_ and H_2_O_2_/Fe^2+^ induced lipid peroxidation more than curcumin (**Table 3**). Among the nine tested compounds, the highest activity was displayed by compound **6** which inhibited lipid peroxidation induced by H_2_O_2_ and H_2_O_2_/Fe^2+^ by 44.4% and 34.1%, respectively. These data are in agreement with the antioxidant activity reported for diarylheptanoids and flavonoids isolated from *C. avellana* cultivar Tonda di Giffoni (Masullo et al., 2015b; Masullo et al., 2016; Cerulli et al., 2017).

## Conclusions

In conclusion, the results obtained in this study allowed to define “Nocciola Piemonte” hazelnut as a good source of polar lipids. In particular, the analysis of the LC-MS profiles of NPF and NPR *n*-butanol extracts allowed to ascertain the occurrence of phospholipids, glycolipids, sphingolipids, and oxylipins, highlighting only small qualitative differences in the metabolite composition between fresh and roasted “Nocciola Piemonte” kernels. Noteworthy was the absence of some l-PA/PA species in NPR and the lack of hydroxylated species of l-PC and PI in NPF, in agreement with that already observed for “Tonda di Giffoni” hazelnut. This finding could be traced back to degradation and oxidation processes caused by the high roasting temperature (Napolitano et al., 2018). This latter seemed to be responsible also for the results obtained by the semiquantitative analysis carried out on the most representative NPR and NPF lipid families, that is, phospholipids and glycolipids, that evidenced the NPR extract as the extract richest in lipids. Indeed, even though lyso- and phospholipids resulted to be the most represented lipid classes in relative terms in both NPR and NPF (with lyso-phospholipids being the prevalent one), the increased levels of the l-PI/PI, l-PC/PC, and l-PG/PG, along with the marked decrease of the l-PA/PA and l-PS levels, both detectable in NPR, could be interpreted as an effect of the roasting temperature. These results are interesting by considering that scientific literature attributes to this lipid classes beneficial effects on human health (Inoue et al., 2011; Napolitano et al., 2018). In particular, PC-rich foods represent good nutrients to improve memory and prevent liver disease in rats, and PI are described as able to give protection in hyperlipidemic deseases and to promote transport, excretion, metabolism, and absorption of the cholesterol in rabbits. For their part, lysoglycerophospholipids are not only structural components of cellular membranes and precursors for the synthesis of glycerolipids but also an essential lipid class as signaling mediators in physiological and pathological processes (Lee et al., 2016; Tan et al., 2020; Hachem and Nacir, 2022).

Furthermore, the phytochemical isolation of phenolics occurring in “Nocciola Piemonte” kernels allowed to unambiguously ascertain, in particular in NPR, the occurrence of flavonoids and giffonins—diarylheptanoids detected in different “Tonda di Giffoni” byproducts and kernels—interestingly highlighting the presence of giffonin L (8) and giffonin V (11), not detected in “Tonda di Giffoni” kernels. These specialized metabolites are known in literature for the antioxidant activity (Bottone et al., 2019; Masullo et al., 2022). In addition, diarylheptanoids from *C. avellana* are reported for their inhibitory activity on human plasma protein carbonylation and oxidation of thiol groups. These results are interesting by considering that protein carbonyl content is used as marker of protein oxidation, which could be responsible for oxidative damage to cells (Masullo et al., 2021b).

In conclusion, the consideration of the synergistic effect of phenolic compounds and bioactive and healthy lipids occurring in the polar fraction of fresh and roasted kernels of “Nocciola Piemonte” concurred to confirm this variety as a precious and beneficial food.

## Data availability statement

The raw data supporting the conclusions of this article will be made available by the authors, without undue reservation.

## Author contributions

SP contributed toward conceptualization, writing, review and editing of the manuscript, supervision, project administration, and funding acquisition. AC, AN, BO, and MM contributed to the methodology, data curation, formal analysis, and writing – original draft preparation. AC and AN contributed to investigation. SP contributed to resources. All authors contributed to the article and approved the submitted version.
